# Arbuscular mycorrhizal symbiosis mitigates the negative effects of salinity on durum wheat

**DOI:** 10.1371/journal.pone.0184158

**Published:** 2017-09-06

**Authors:** Veronica Fileccia, Paolo Ruisi, Rosolino Ingraffia, Dario Giambalvo, Alfonso Salvatore Frenda, Federico Martinelli

**Affiliations:** Dipartimento di Scienze Agrarie, Alimentari e Forestali, Università degli Studi di Palermo, Palermo, Italy; University of Western Sydney, AUSTRALIA

## Abstract

Arbuscular mycorrhizal (AM) symbiosis is generally considered to be effective in ameliorating the plant tolerance to salt stress. Unfortunately, the comprehension of the mechanisms implicated in salinity stress alleviation by AM symbiosis is far from being complete. Thus, an experiment was performed by growing durum wheat (*Triticum durum* Desf.) plants under salt-stress conditions to evaluate the influence of AM symbiosis on both the plant growth and the regulation of a number of genes related to salt stress and nutrient uptake. Durum wheat plants were grown outdoors in pots in absence or in presence of salt stress and with or without AM fungi inoculation. The inoculum consisted of a mixture of spores of *Rhizophagus irregularis* (formerly *Glomus intraradices*) and *Funneliformis mosseae* (formerly *G*. *mosseae*). Results indicate that AM symbiosis can alleviate the detrimental effects of salt stress on the growth of durum wheat plants. In fact, under salt stress conditions mycorrhizal plants produced more aboveground and root biomass, had higher N uptake and aboveground N concentration, and showed greater stability of plasma membranes compared to non-mycorrhizal plants. Inoculation with AM fungi had no effect on the expression of the N transporter genes AMT1.1, AMT1.2, and NAR2.2, either under no-stress or salt stress conditions, probably due to the fact that plants were grown under optimal N conditions; on the contrary, NRT1.1 was always upregulated by AM symbiosis. Moreover, the level of expression of the drought stress-related genes AQP1, AQP4, PIP1, DREB5, and DHN15.3 observed in the mycorrhizal stressed plants was markedly lower than that observed in the non-mycorrhizal stressed plants and very close to that observed in the non-stressed plants. Our hypothesis is that, in the present study, AM symbiosis did not increase the plant tolerance to salt stress but instead generated a condition in which plants were subjected to a level of salt stress lower than that of non-mycorrhizal plants.

## Introduction

Soil salinity is one of the most serious environmental stresses that limit crop production; more than 6% of the world’s total land area is indeed affected by salinity and sodicity [1]. High concentrations in soil of cations such as sodium (Na^+^) or anions such as chloride (Cl^–^) make it difficult for plant roots to extract water (due to the reduction of soil osmotic potential) and nutrients, and high concentrations of salts inside the plant can have toxic effects [1], with inhibition of protein synthesis, disruption of enzymes, damage of membrane integrity and cell organelles [2]. To avoid damages from salinity, plants have evolved several mechanisms that are implicated in ionic and water/osmotic homeostasis through the regulation of genes involved in the transport and compartmentation of nutrients [3], the accumulation of solutes [4], and the expression of aquaporins [5], the latter being a group of water-channel proteins that promote and regulate the passive movement of water molecules through a water potential gradient [6–7]. However, although it is well established that aquaporins play an important role in regulating the transcellular transport of water in plant tissues, the comprehension of the relationship between expression of aquaporin genes and plant response to water deficit caused by osmotic stress still remains quite limited. Besides these mechanisms, plants have also evolved systems to repair the cellular damages caused by salinity. For instance, changes in the expression of dehydrin (DHN) genes in plants grown under water-related stresses have been reported [8–9]. Dehydrins are considered as stress proteins involved in formation of plant protective reactions against dehydration, but their specific function has not been well understood so far [10–11]. [12] showed that *Arabidopsis thaliana* (L.) Heynh overexpressing the wheat dehydrin DHN-5 maintained higher reactive oxygen species (ROS)-scavenging enzymatic activity and accumulated lower levels of hydrogen peroxide (H_2_O_2_), thus improving their resistance to water-related stress.

In addition to these intrinsic mechanisms of adaptation, plants growing under adverse environmental conditions, such as saline soils, can improve their performance indirectly, by establishing associative relationships with a number of soil microorganisms, such as bacteria and/or fungi. Among the fungi, arbuscular-mycorrhizal (AM) fungi are able to activate symbiotic relationships with the majority of land plants. AM symbiosis has a positive influence on plant growth, which is mainly attributable to the ability of AM fungi to take up from the soil both water [13–14] and nutrients—especially phosphorus (P) [15–16], and to a lesser extent nitrogen (N) [17]—and deliver them to the roots of its host, and also to enhance the health of its host by protecting it from pathogens, pests, and parasitic plants [18]. AM fungi occur naturally in saline soils and they can be present even under severe salinity [19–20]. Many studies have shown that AM symbiosis can improve the tolerance of plants in growing under salt-stress conditions, reducing their yield losses [21–25]. Several mechanisms have been suggested to be involved in the increased salinity tolerance of mycorrhizal plants compared to non-mycorrhizal plants, including enhanced capacity in the uptake of both water and nutrients—mainly P, N, calcium (Ca), and potassium (K) [24,26]—, better maintenance of membrane integrity (which facilitates compartmentation of Na^+^ and Cl^−^within vacuoles and selective ion intake and translocation) [27], maintenance of proper K^+^/Na^+^ ratios in plant tissues (thus helping to prevent the disruption of K-mediated enzymatic processes and the inhibition of protein synthesis) [28–29], enhanced osmoregulation due to a higher accumulation of osmoprotectant solutes—such as proline and glycine betaine—and soluble sugars in plant tissues [25,30]. However, according to [2], the understanding of the deeper mechanisms that allow mycorrhizal plants to exhibit higher tolerance to salinity is far from being complete. In particular, the molecular mechanisms involved in this beneficial effect are still poorly investigated. Consequently, to improve the comprehension of the mechanisms implicated in salinity stress alleviation by AM symbiosis, it is relevant to study how the expression of the genes involved in the regulation of functions such as the uptake and transport of water and nutrients varies in mycorrhizal plants grown under salt-stress conditions. Hence, an experiment was conducted growing durum wheat (*Triticum durum* Desf.) plants under salt-stress conditions to evaluate the influence of AM symbiosis on the expression of a number of genes (nitrate and ammonium transporters and drought stress-related genes) most likely of relevance in plant response to salinity stress. Moreover, the agronomic response to salinity of durum wheat mycorrhizal plants was also evaluated to find relationships with the variation in gene expression. Durum wheat was chosen as model plant for this investigation due to its importance as crop plant in the arid and semiarid areas of the Mediterranean basin.

## Materials and methods

### Ethics statement

No specific permits were required for the described study. The experiment did not involve endangered or protected species.

### Plant material and experimental design

Durum wheat plants were grown outdoors in pots under four conditions: absence of salinity stress with or without AM fungi inoculation (‘No-stress +AM’ and ‘No-stress–AM’, respectively); presence of salinity stress with or without AM fungi inoculation (‘Saline-stress +AM’ and ‘Saline-stress–AM’, respectively). A complete randomized factorial design was adopted considering seven replicates. Each pot (diameter 150 mm, height 130 mm) was filled with 2000 g of a quartz sand:soil mixture (1:1). Soil properties were as follows: 267 g kg^–1^ clay, 247 g kg^–1^ silt, and 486 g kg^–1^ sand; pH 8.0; 6.3 g kg^–1^ total carbon (C); 0.86 g kg^–1^ total N; 1.70 dS m^–1^ saturated electrical conductivity (EC) (25°C). Both soil and sand were sieved through a 2 mm mesh and autoclaved at 121°C for 20 min in order to completely impair soil biological (both fungal and bacterial) activity. The bacterial microflora was extracted by suspending 500 g soil in 1.5 L distilled water. After shaking and decanting, the suspension was filtered (11 μm mesh) to discard natural AM fungi. Before starting the experiment, each pot received 30 ml of soil suspension filtrate to reintroduce the natural microbial community. Inoculation with AM fungi involved the application of a commercial AM inoculum at a rate of 10 g per pot. The inoculum consisted of a mixture of spores of *Rhizophagus irregularis* (formerly *Glomus intraradices*) and *Funneliformis mosseae* (formerly *G*. *mosseae*), each of which was present at a rate of 700 spores g^–1^ of inoculum. Each pot received 60 mg of N in the form of ammonium sulfate ([NH_4_]_2_SO_4_).

Sixteen seeds of durum wheat (cv. Anco Marzio), previously surface-sterilized with H_2_O_2_ at 4% for 3 minutes, were sown in each pot. Ten days after emergence, plants were thinned to six seedlings per pot. To avoid the negative effect of salinity on both the thin seedlings and the establishment of the AM symbiosis, wheat plants were grown for 15 days before the application of the salinity treatment. The latter was obtained by adding NaCl in irrigation water (0 and 10 g L^–1^). To prevent osmotic shock, salt was added gradually by distributing in total 1 L of the NaCl solution in each pot during the 7 days starting from the beginning of the salinity treatment. This led the EC of saturated soil extract to 1.50 and to 13.00 dS m^–1^ in the non-stressed and salt-stressed treatments, respectively. From this moment, plants were watered with tap water (0.58 dS m^–1^) until harvest. Leaching was avoided by maintaining soil water always below field capacity. During the experiment, irrigation was done every two days and, for each pot, the amount of irrigation water consisted of total replenishment of water lost though evapotranspiration. Evapotranspiration losses were determined considering the variations in pot weight measured daily.

All pots were harvested after 45 days from sowing. On the harvest day, before biomass was sampled, the chlorophyll contents of leaves were determined using a hand-held chlorophyll meter (SPAD-502, Konica Minolta, Osaka, Japan), averaging readings from ten full expanded leaves of plants randomly selected in each pot. After this, plant biomass was immediately separated into roots, stems, green leaves, and senescent and dry leaves, and fresh weights were recorded. About 1 g of green leaves and 1 g of roots from each pot were immediately frozen in liquid N, stored at −80°C, and subsequently pulverized without thawing. At the same time, a sample of green full expanded leaves (about 400 mg) was taken from each pot to determine the membrane stability index (MSI). The leaf material was divided in two sets of 200 mg each. The first set was heated at 40°C for 30 min in a water bath (10 cm^3^); then the electrical conductivity bridge (C1) was measured. The second set was boiled at 100°C for 10 min (in 10 cm^3^ of water) before measuring the electrical conductivity bridge (C2). MSI was calculated according to the formula by [31]:

$$MSI=\left[1-\frac{C1}{C2}\right]\times 100$$

Moreover a representative root sample (about 1 g) was taken from each pot to determine the overall colonization of roots by AM fungi. To this end, root samples were cleared with 100 g L^–1^ potassium hydroxide (KOH) and stained with 50 mg L^–1^ trypan blue following the method described by [32]. Root colonization by AM fungi was then measured with the grid intersect method according to [33].

For each pot, the remaining plant biomass was dried at 65°C for 36 h (separately for each botanical fraction) to determine the dry matter content and calculate the belowground and aboveground dry masses. Moreover, plant N content was determined separately for each botanical fraction using the combustion method of Dumas (DuMaster D-480, Büchi Labortechnik AG, Flawil, Switzerland). For each pot, total N uptake was calculated as the sum of N accumulated in roots (root dry mass × root N concentration) and shoots (shoot dry mass × shoot N concentration).

### RNA extraction and cDNA preparation

RNA was extracted using the Spectrum Plant Total RNA kit (Sigma). RNA quantity was measured with the Nanodrop and the quality was analyzed using electrophoresis by loading 1 μL of sample on 2% agarose gel. DNase treatment and cDNA synthesis were performed in a combined protocol following Quantitect Reverse Transcription Kit (Qiagen) instructions. For each treatment, three biological replicates were considered, each made of a pool of healthy fully expanded leaves taken from all the plants in the pot.

### Gene expression analysis

Quantitative RT-PCR was used to analyze the expression of durum wheat genes. Eleven genes were analyzed belonging to nitrate and ammonium transporters (NRT1.1, NAR2.2, AMT1.1, and AMT1.2) and drought stress-related genes (AQP1, AQP4, PIP1, NAC8, DREB5, DREB6, and DHN15.3). For each target gene, PCR primers were designed basing on *T*. *aestivum* sequences deposited in NCBI (https://www.ncbi.nlm.nih.gov/genbank/; Table 1). Real Time PCR was performed with iTaq Universal SYBR Green Supermix (BioRad). Amplifications were conducted using 25 ng cDNA in a 15 μL final volume with a BioRad iQ5 PCR system (BioRad) with standard conditions: 3 min at 95°C, 40 cycles of 15 s at 95°C, and 45 s at 60°C. All PCR reactions were performed in duplicates. The 18S of *T*. *aestivum* was used as an endogenous reference gene. The qPCR results were analyzed using the 2^−ΔΔ*C*T^ method [34]. Fluorescent signals were collected during the annealing step and the cycle threshold (*C*_T_) values extracted with an auto-calculated threshold followed by baseline subtraction. Relative changes in expression were determined by calculating the ΔΔ*C*_T_ between the target (*C*_T_ sample) and reference (*C*_T_ 18S) genes.

**Table 1:** List of primers used in qRT-PCR analysis.

Gene	GenBank Accession number	Primer sequences
**NRT1.1**	AY587265.1	F: CACAGCGAATAGGGATTGGT
		R: CGCCTAGCAGGAAGTACTGG
**NAR2.2**	AY763795.1	F: CCTCTCCAAGCTTCCTGTGA
		R: CGTAGCAGAGGCTGACCTT
**AMT1.1**	AY390355.1	F: CCAAGAACACCATGAACATC
		R: GGAAGAGGAAGAAGCTGTAG
**AMT1.2**	AY525638.1	F: CGGCTTCGACTACAGCTTCT
		R: AGTGGGACACCACAGGGTAG
**AQP1**	DQ867075.1	F: AGCGAACAAGTACTCGGAG
		R: TAGAGGAAGAGGGAGGTG
**AQP4**	DQ867078.1	F: CGGATGTGGTCCTTCTAC
		R: ACGAGGACGAAGATCATG
**DHN15.3**	AM180931.1	F: CGTCGACGAGTACGGTAAC
		R: CCATGCCATCATCCTCAGAC
**PIP1**	AF366564.1	F: CACCTTCGGGCTGTTTTTG
		R: GTCTGGAACCCCTTGACC
**NAC8**	HM027573.1	F: CGCATGGGATGATGTCAAG
		R: CATAGGGAAGTTCACCGTC
**DREB5**	AY781358.1	F: GAGGAACTTGTGGAGCAGAG
		R: ATCTCCGAGGTCGCTTTTTC
**DREB6**	AY781361.1	F: AAAACCAGAAGCTCCTGC
		R: TGCTCTGAGAAGTTGACAC
18S*	AB778770.1	F: CAACGGATATCTCGGCTCTC
		R: TTGCGTTCAAAGACTCGATG

*Ribosomal 18S of *T*. *aestivum* was used as endogenous reference.

### Statistical data analysis

The collected data were subjected to analysis of variance (ANOVA) according to the experimental design. Variables corresponding to proportions were arcsine transformed before analysis to assure a better fit with the Gaussian law distribution. Treatment means were compared using Fisher’s protected least significant differences test at the 5% probability level. Principal component analysis (PCA) was performed using data on gene expression to determine i) whether multivariate differences existed in plant response to the applied treatments (‘Salinity stress’ and ‘Mycorrhizal inoculation’) and ii) which genes accounted the most for these differences. Centroid values and their standard errors were calculated for each combination (‘Salinity stress × Mycorrhizal inoculation’). The SAS software [35] was used for all statistical analyses.

## Results

### Response of wheat plants exposed to salinity

Salinity significantly affected all the traits measured in plants (Tables 2 and 3). On average, compared to the control (non-stressed condition), salt stress reduced the number of stems per plant (–47%), the aboveground biomass (–33%), and, particularly, the root biomass (–64%; Table 2). On the contrary, plants grown under salt stress conditions had chlorophyll meter readings (SPAD values) higher than non-stressed plants. Similarly, the N concentrations of both the root and the aboveground biomass (the latter including green leaves, senescent and dry leaves, and stems) were all significantly higher under salt stress compared to the control (Table 3). On the contrary, the total N uptake was on average markedly lower in salt stressed plants (–30%).

**Table 2:** Number of stems per plant, aboveground and root biomass (as grams of dry matter per pot), proportion of green leaves, SPAD value, membrane stability index (MSI), and levels of mycorrhizal infection in durum wheat grown under no- and saline-stress regimes and in the presence or absence of arbuscular mycorrhizal symbiosis. +AM = inoculation with arbuscular mycorrhizal spores;–AM, suppression of arbuscular mycorrhizal symbiosis.

Trait		No-stress		Saline-stress		Significance		
		+AM	–AM	+AM	–AM	Stress	Inoc.	Stress × Inoc.
No. stems per plant	n°	4.6	5.0	2.7	2.4	***	*ns*	*ns*
Aboveground biomass (AB)	g per pot	2.19	2.15	1.51	1.39	***	*	*ns*
Root biomass	g per pot	2.33	2.14	0.93	0.70	***	*	*ns*
Proportion of green leaves	% on AB	50.1	46.2	34.3	34.1	***	*ns*	*ns*
SPAD value	—	50.5	49.3	53.1	52.1	***	*	*ns*
MSI	—	86.1	86.0	75.0	66.6	***	*ns*	*
Mycorrhizal infection	%	36.4	0.8	31.2	0.5	*	***	*ns*

***, **, * denote significant differences at 0.001, 0.01, and 0.05 probability levels, respectively; *ns* denotes differences not significant.

**Table 3:** Nitrogen concentration in the aboveground (total and separately for each botanical fraction) and root biomasses, and total N uptake in durum wheat grown under no- and saline-stress regimes and in the presence or absence of arbuscular mycorrhizal symbiosis. +AM = inoculation with arbuscular mycorrhizal spores;–AM, suppression of arbuscular mycorrhizal symbiosis.

Trait		No-stress		Saline-stress		Significance		
		+AM	–AM	+AM	–AM	Stress	Inoc.	Stress × Inoc.
N concentration of:								
Total aboveground biomass	g kg^–1^	30.9	26.5	36.5	33.1	***^˟^	***	*ns*
*Green leaves*	g kg^–1^	38.5	34.3	40.9	38.9	***	***	*
*Senescent and dry leaves*	g kg^–1^	20.4	20.9	33.8	35.9	***	*ns*	*ns*
*Stems*	g kg^–1^	25.1	21.2	27.4	23.2	*	***	*ns*
Root biomass	g kg^–1^	12.4	12.2	14.3	15.0	**	*ns*	*ns*
Total N uptake	mg N per pot	95.1	82.6	68.5	56.3	***	***	*ns*

^˟^***, **, * denote significant differences at 0.001, 0.01, and 0.05 probability levels, respectively; *ns* denotes differences not significant.

Uninoculated plants showed insignificant mycorrhizal colonization levels (always <1% of root length colonized; Table 2). Characteristic structures of AM fungi were observed in the roots after inoculation, with mycorrhizal colonization levels >30% both in non-stressed and salt stressed conditions. On average, compared to the non-mycorrhizal treatment, AM plants showed higher aboveground and root biomass (+5% and +14%, respectively), whereas no effect of AM symbiosis was observed on the number of stems per plant and the proportion of leaves on the aboveground biomass. Mycorrhizal plants showed SPAD values slightly higher than uninoculated plants under both salt stressed and non-stressed conditions. Moreover, AM plants, compared to non-AM plants, had a higher total N uptake (+18% on average) and a higher N concentration in the total aboveground biomass (+13% on average; Table 3). This evidence was confirmed for all the botanical fractions except for the roots. The effects of both the treatments applied (‘Salinity stress’ and ‘Mycorrhizal inoculation’) on SPAD values were similar to those observed for total aboveground biomass N concentration. These two traits were strictly and positively correlated (S1 Fig).

Soil salinization significantly decreased MSI values compared to the non-stressed condition (–18% on average; Table 2). The interaction ‘Salinity stress × Mycorrhizal inoculation’ was significant at the 5% probability level; in fact, while under non-stressed conditions no effect was observed by AM symbiosis, under salinity stress the MSI values were significantly higher in +AM compared to–AM treatment.

### Nitrogen transporters and stress-related genes

**Fig 1:**
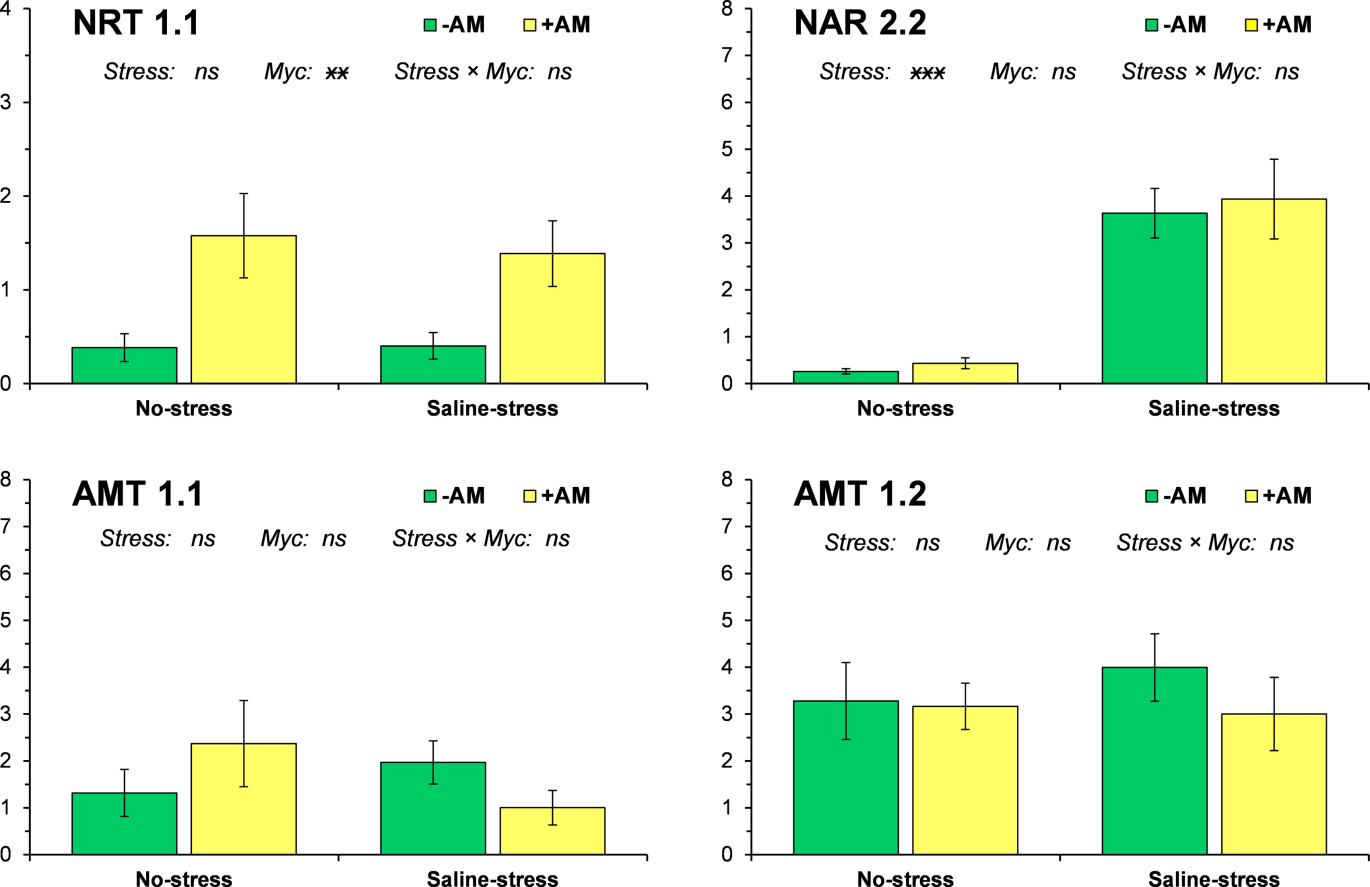
Expression of nitrogen transporter genes NRT1.1, NAR2.2, AMT1.1 and AMT1.2 in response to salinity stress and mycorrhizal inoculation. Vertical bars indicate ± standard error of each mean value. For each gene, ***, **, *, and † denote significance at 0.001, 0.01, 0.05, and 0.1 probability levels, respectively; *ns* denotes not significant at 0.1 probability level.

**Fig 2:**
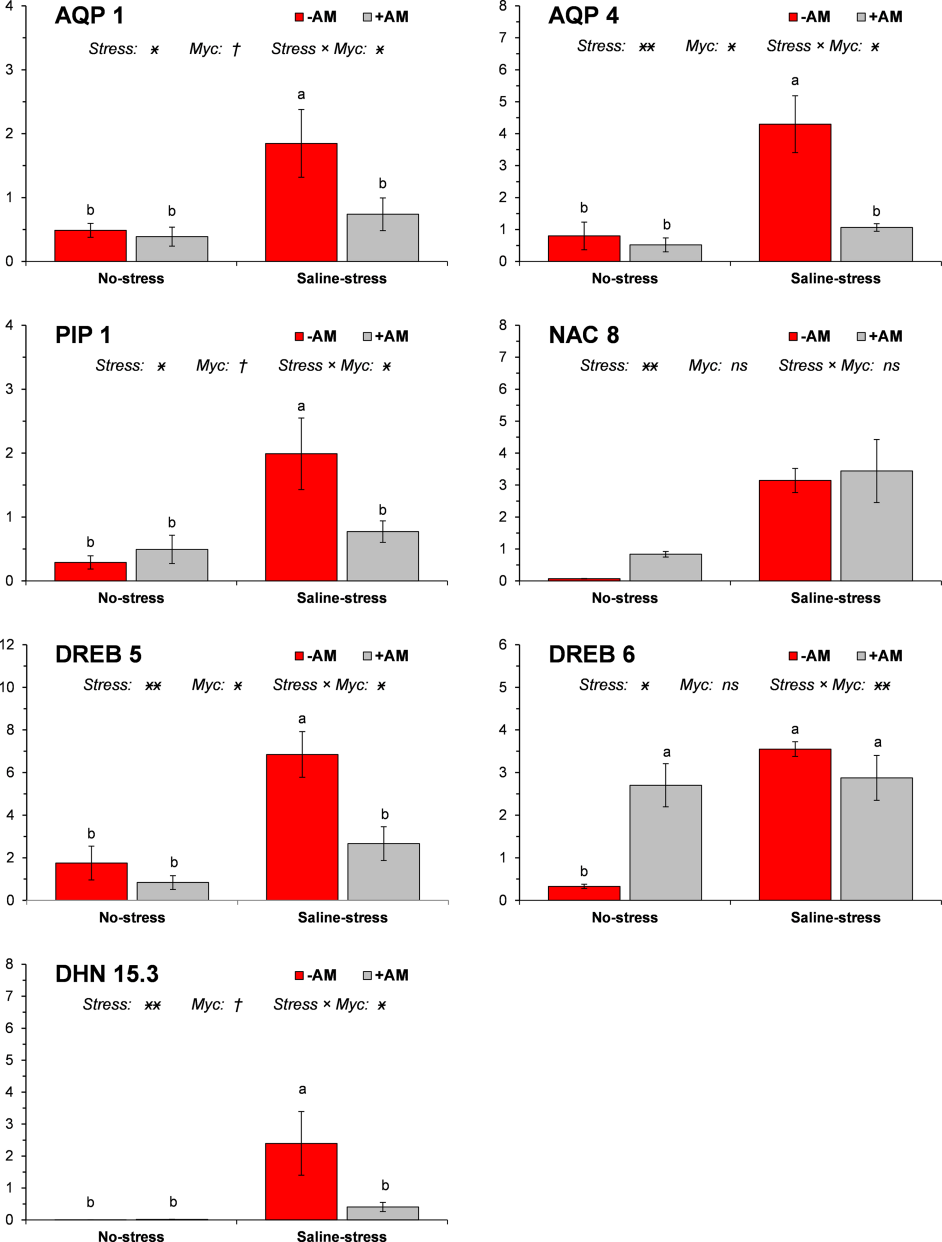
Expression of drought stress-related genes AQP1, AQP4, PIP1, NAC8, DREB5, DREB6, and DHN15.3 in response to salinity stress and mycorrhizal inoculation. Vertical bars indicate ± standard error of each mean value. For each gene, ***, **, *, and † denote significance at 0.001, 0.01, 0.05, and 0.1 probability levels, respectively; *ns* denotes not significant at 0.1 probability level. Different letters denote significant differences at 0.05 probability level.

Salinity markedly increased the expression of NAR2.2 whereas mycorrhizal inoculation significantly upregulated NRT1.1 (Fig 1). The expression of both the ammonium transporters (AMT1.1 and AMT1.2) was significantly affected neither by salinity nor by mycorrhizal symbiosis. All stress-related genes (AQP1, AQP4, PIP1, NAC8, DREB5, DREB6, and DHN15.3) were upregulated by saline stress (Fig 2). Mycorrhizal symbiosis significantly mitigated the salt-induction of all these genes except NAC8. Under no-stress conditions the inoculation with AM fungi determined an upregulation of only DREB6 among the stress-related genes.

### Principal component analysis

**Fig 3:**
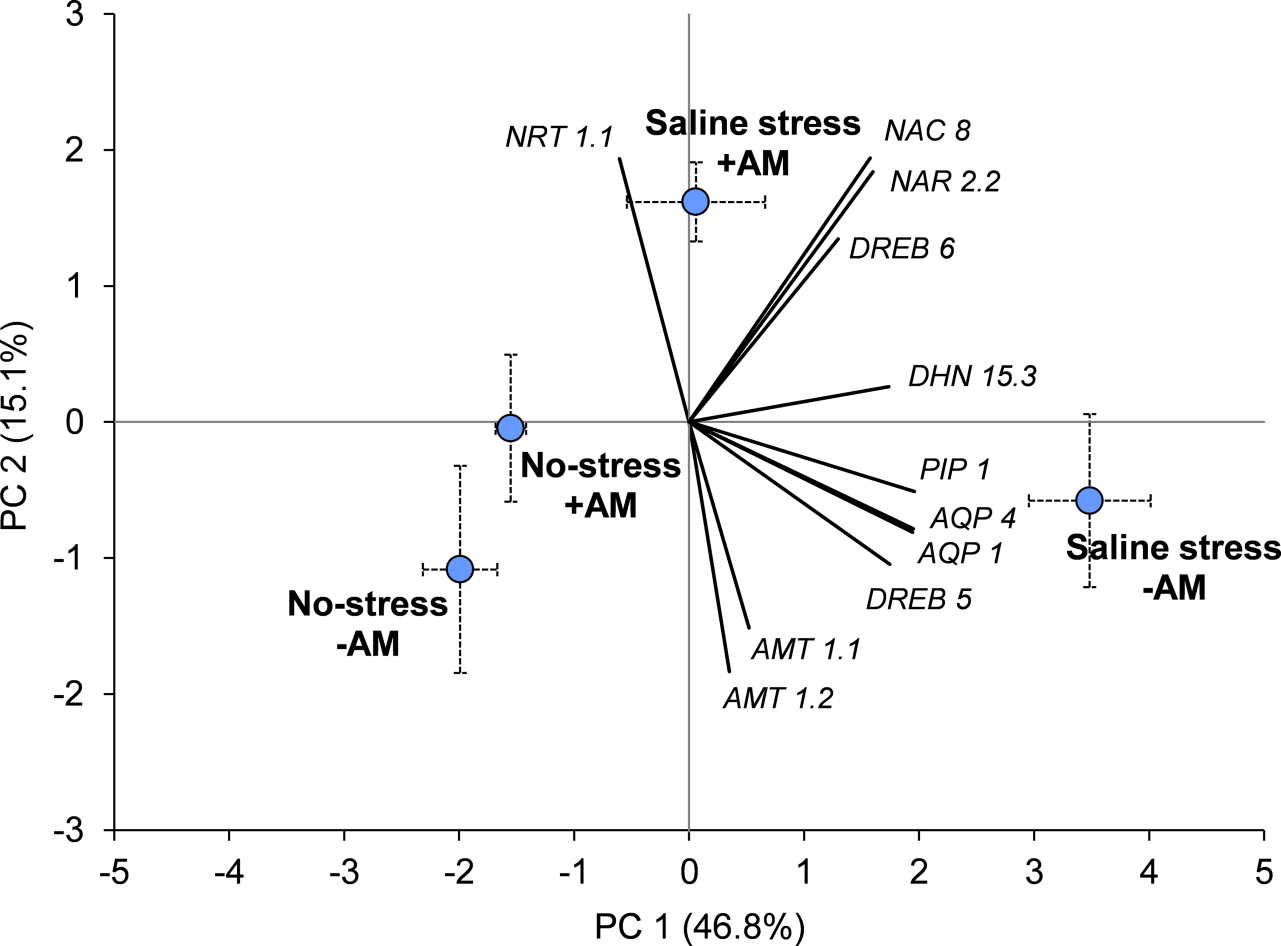
Principal component analysis of the four salt stress conditions of durum wheat in presence and absence of both salinity and mycorrhizal inoculation. Components of the eleven analyzed genes were also indicated. No-stress +AM and No-stress–AM means absence of salt stress with or without AM fungi inoculations, respectively; Saline-stress +AM and Saline-stress–AM means presence of salt stress with or without AM fungi inoculations, respectively.

Principal component analysis (PCA) based on gene expression data clearly discriminated the four ‘Salinity stress × Mycorrhizal inoculation’ combinations (Fig 3). ‘No-stress +AM’ and ‘No-strees–AM’ plotted near each other (bottom left quadrant) but distant from both ‘Saline-stress +AM’ (upper right quadrant) and ‘Saline-stress–AM’ (bottom right quadrant). The PC1 accounted for about 47% of the total variation and varied mainly according to PIP1, AQP4, and AQP1. The PC2 accounted for 15% of the total variation and was influenced mainly by nitrogen transporter genes (NAR2.2, NRT1.1, and AMT1.2) and by NAC8.

## Discussion

Soil salinity is an environmental stress that drastically affects crop growth and productivity. Many studies have demonstrated that salinity can inhibit plant growth through several mechanisms including damage of enzymes and plasma membranes [36], reduction in plant water availability (due to the lower soil water potential) [1], accumulation of toxic elements (i.e., Na^+^ and Cl^–^), inhibition of chlorophyll and protein synthesis, reduction in nutrient uptake, transport and/or partitioning within the plant [37]. In the present study, a severe reduction in both shoot and root biomass was observed in plants of durum wheat when salinity stress was imposed at tillering stage. This reduction was associated to decreases in the stability of membranes (< MSI values in salt stressed plants) and the total N uptake. Many studies showed that salinity can reduce N accumulation in crop plants and that this decrease is generally accompanied by an increase in Cl^−^uptake. Therefore, the decrease in plant N uptake is probably to be partially related to the antagonism of nitrate metabolism from chloride [38]. However, in the present study, while the total N accumulated by plants decreased under salt stress conditions, the N concentrations in all plant tissues (leaves, stems, roots) increased. This result may seem surprising, as much research reports decreases in plant tissues N concentrations due to the negative effects of salt stress on plant N uptake [39]. Nevertheless, other studies have shown that the plant N concentration increases or remains unchanged when plants are grown under optimal N conditions [40–41]. The latter condition certainly occurred in the present research in which N was not a limiting factor thanks to the substrate chemical characteristics and the amount of N-fertilizer applied. Therefore, our findings suggest that the negative effects of salinity observed on plant growth cannot be attributed to difficulties in N absorption. At the same time, it is interesting to highlight how the effects of salt stress affected the efficiency of N transport within plant tissues. In particular, a considerable enhancement of the expression of NAR2.2 was observed. The latter is a protein that actively interacts with genes of the NRT2 family to form a functional high-affinity transport systems (HATS) effective in NO_3_^–^ transport [42], whose absence, as seen in *Arabidopsis*, is associated with marked reductions of leaf N content [43]. Therefore, the higher N concentration observed in leaves of plants grown under saline conditions compared to control seems somehow linked to an upregulation of this gene. The high N concentration values observed in the senescent and dry leaves of plants grown under salt stress conditions (which were close to those of green leaves) demonstrate that salt stress prevented the translocation of this element from the senescent leaves to the other plant organs (differently from what observed in plants not subjected to salt stress). On the other hand, salinity may have a negative effect on membrane proteins and change their integrity [44], thus compromising nutrient absorption and translocation to the different organs.

Under non-stressed conditions, wheat plants inoculated with AM fungi accumulated more N than non-mycorrhizal plants. This result could be attributable to both the facilitation in host N uptake by AM fungi through the extensive extraradical hyphal network that increases the volume of soil explored by mycorrhizal plants compared to non-mycorrhizal plants [45], and the enhanced effectiveness of AM plants than roots alone in competing with soil microorganisms for inorganic N [46]. The increased N accumulation in leaf tissues may be also partially due to the AM symbiosis-upregulation, in both stressed and non-stressed conditions, of N transport genes such as NRT1.1. This gene is a dual-affinity transporter that contributes to both low- and high-affinity nitrate uptake in roots [47–49]. In addition to nitrate uptake, NRT1.1 is also involved in young leaves development [50], stimulation of lateral root proliferation [51], light-induced stomatal opening [52], repression of NRT2.1 [53], stimulation of reproductive growth, affecting flower timing and flower bud expansion [50]. This set of functions leads to hypothesize that this gene may act as a nitrate sensor or signal transducer [54]. Moreover, this gene leads to the import of nitrate in leaf tissues from the apoplast (xylem) and the induction of this gene may contribute to the increase of N uptake observed in leaf tissues. Thus, the higher leaf and stem N concentration in mycorrhizal plants might be due to mycorrhizal-driven transcriptional activation of genes encoding N transport. In the present study no effects of AM symbiosis were found on the expression of the other N transporter genes investigated (AMT1.1, AMT1.2, and NAR2.2), either under no-stress or saline stress conditions. [55] observed in durum wheat that the expression of NRT1.1, NRT2, NAR2.2, AMT1.2, and AMT2.1 were significantly upregulated by mycorrhizal symbiosis but only when plants were grown under N-limiting conditions. Probably, in the present study, the lack of an effect of mycorrhizal symbiosis on the expression of the N transporter genes (with the exception of NRT1.1) was attributable to the fact that durum wheat plants were grown, as said, under optimal N conditions.

Data from the present study revealed that AM symbiosis can mitigate the negative effects of salt stress on plant growth. In particular, under salt stress conditions AM symbiosis had a favorable impact on N acquisition and N concentration, and aboveground and root biomass, in agreement with the findings of many studies, as reviewed by [56]. Moreover, we detected a clear positive effect of AM symbiosis on the alleviation of the damaging effect of salinity on the stability of plasma membranes.

Salinity stress has similar effects of a water-deficit condition making water acquisition more difficult for the plant [57]. Thus it is generally accepted that plants regulate the expression of the drought stress-responsive genes to respond to salt stress. In the present experiment, all the drought-regulated genes investigated were induced under salt stress condition. Our results are in agreement with those of many studies in which emerged that salinity induces higher expression of aquaporin genes, DREBs (dehydration responsive element binding) and NAC transcription factors [58–60]. Furthermore, other researches provide evidence for a positive correlation between DHN gene expression and abiotic stress tolerance of many plant species including durum wheat [61–63].

Under saline stress conditions, wheat plants inoculated with AM fungi showed a markedly lower expression of almost all the drought stress-related genes (AQP1, AQP4, PIP1, DREB5, and DHN15.3) compared to the non-mycorrhizal plants. This result is in contrast to what is generally reported in the literature. Many studies, in fact, showed that the mycorrhizal symbiosis was generally associated with an increase in the expression of some aquaporins (in the root tissues) when plants were grown under salt stress conditions [5,64–65]. The authors of these works speculated that the upregulation of aquaporin genes was one of the mechanisms through which the mycorrhizal symbiosis enhances the regulation of the plant water status, so contributing to the plant tolerance to the stress conditions generated by salinity. In the present study, the level of expression of almost all the investigated drought stress-related genes in the mycorrhizal stressed plants was closer to that observed in the non-stressed plants compared to that observed in non-mycorrhizal stressed plants. This is also clearly evident by the analysis of the PCA diagram. Our data, therefore, seem to indicate that mycorrhizal symbiosis reduces the need for the plant to activate mechanisms of response to salt stress. This suggests that mycorrhizal plants are subjected to a lower level of stress caused by salinity compared to non- mycorrhizal plants. This hypothesis is very intriguing and certainly deserves further research that should be directed to the understanding of the mechanisms through which the mycorrhizas improve the plant water uptake and the water flow through host plants under both drought and salinity conditions. However, it must be kept in mind that a plurality of mechanisms (that do not exclude one another but which often interact with each other) brings the AM symbiosis to alleviate the saline stress in the host plant. It should also be remembered that a correct reading of our data cannot fail to consider that the regulation of plant abiotic stress response genes may vary greatly depending on a number of factors: salinity and duration of plant exposure to it, type of abiotic stress, investigated genes, plant and AM fungus species, plant tissue, etc.

## Supporting information

S1 Fig Relationship between the aboveground N concentration and the SPAD values. The traits were measured in durum wheat grown under no- and saline-stress and in the presence or absence of arbuscular mycorrhizal symbiosis.
(PDF)
