# Supplementary material for: Arbuscular mycorrhizal symbiosis mitigates the negative effects of salinity on durum wheat
Source: PLoS One. 2017 Sep 6;12(9):e0184158. doi: 10.1371/journal.pone.0184158 (PMC5587292; doi:10.1371/journal.pone.0184158)
Supplement: S1 Fig — The traits were measured in durum wheat grown under no- and saline-stress and in the presence or absence of arbuscular mycorrhizal symbiosis. (PDF) [file pone.0184158.s001.pdf]

Supporting information

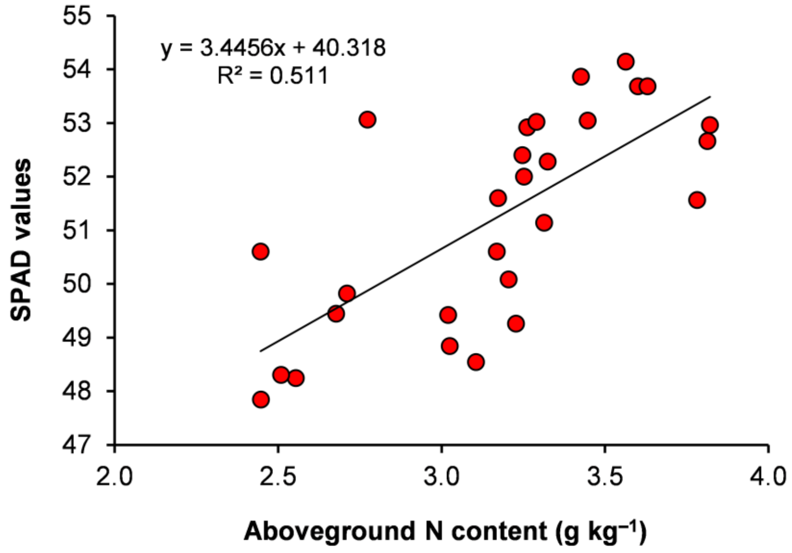

S1 Fig. Relationship between the aboveground N concentration and the SPAD values. The traits were measured in durum wheat grown under no- and saline-stress and in the presence or absence of arbuscular mycorrhizal symbiosis.
